# Sustainable Development Solutions: Growing and Processing Raspberries on Lithuanian Farms

**DOI:** 10.3390/foods12213930

**Published:** 2023-10-26

**Authors:** Audrone Ispiryan, Algirdas Giedraitis, Kristina Sermuksnyte-Alesiuniene, Marian Butu, Vilma Atkociuniene, Alina Butu, Jonas Viskelis, Astrida Miceikiene

**Affiliations:** 1 Institute of Horticulture, Lithuanian Research Centre for Agriculture and Forestry, Instituto al. 1, LT-58344 Akademija, Lithuania; jonas.viskelis@lammc.lt; 2 Faculty of Social Sciences and Humanities, Klaipeda University, 84 Herkus Mantas Str. 84, LT-92294 Klaipėda, Lithuania; giedraitis.algirdas@gmail.com; 3 Lithuanian Centre for Social Sciences, Institute of Economics and Rural Development, A. Vivulskio Str. 4A-13, LT-03220 Vilnius, Lithuania; kristina@agrifood.lt; 4 National Institute of Research and Development for Biological Sciences, Splaiul Independentei 296, 060031 Bucharest, Romania; marian_butu@yahoo.com (M.B.); alina_butu@yahoo.com (A.B.); 5 Research Institute for Agriculture Economy and Rural Development, Bd Marasti 61, 010961 Bucharest, Romania; 6 Agriculture Academy, Vytautas Magnus University, Studentų Str. 11, LT-53361 Akademija, Lithuania; vilma.atkociuniene@vdu.lt (V.A.); astride.miceikiene@vdu.lt (A.M.)

**Keywords:** food loss and waste, raspberry products, value-added cultivation and processing, holistic management and economy, sustainable, digital transformation

## Abstract

The EU’s goals by 2050 are to ensure food security, prevent bio-diversity loss, and strengthen the EU food system’s resilience. Recent scientific research and the situation in the global market show that the cultivation and processing of raspberries is currently completely unsustainable. This sector is experiencing a huge decline in Lithuania. Therefore, we chose the sustainability of raspberry growing (from farm) and processing (to fork) as an object. The aim of this article was (i) to analyze the raw material of the raspberry plant for product sustainable processing, (ii) to create a digital sustainability measurement model, and (iii) to present sustainable development solutions for effective raspberry growing and processing on Lithuanian farms using content and descriptive methods. This paper discusses how to help small raspberry growers and processors achieve sustainable economic, environmental, and social performance from field raw material to processed products. Analysis of the scientific literature has revealed qualitative and quantitative sustainability indicators for improving raspberry production. The assessment of the sustainability according to our created model revealed the (un)sustainable factors and the current situation in raspberry farms on a Likert scale from very unsustainable to very sustainable. Based on the evaluation we have determined sustainable development solutions. Raspberry growing and processing in Lithuania can contribute to environmental conservation, economic growth, and social well-being, fostering a more sustainable and resilient agricultural sector by investing in R&D, improving productivity, creating employment opportunities and supporting rural communities, establishing a robust waste management system, and embracing renewable energy sources. Raspberry growers and processors can use the digital model we created for the sustainability, efficiency, and development directions of their farm.

## 1. Introduction

In the modern conditions of economic, environmental, and social development, as hallmarks of scientific and technological progress, a number of new phenomena and circumstances have emerged. Understanding and responding to them inevitably leads to the need to delve into the so-called sustainable development problems and strive to solve these problems adequately to challenge the new ones arising in society [1,2,3]. The importance and significance of sustainable development issues are shown by the fact that understanding and solving these problems is one of the main priorities implemented in modern scientific research practice. A shift to an effective sustainable food system can bring environmental, health, and social benefits, as well as offer fairer economic gains. The international community is currently on track to realize the Sustainable Development Goals (SDGs) [4,5,6,7,8].

The aim of this article was (i) to analyze the raw material of the raspberry plant for product sustainable processing, (ii) to create a digital sustainability measurement model, and (iii) to present sustainable development solutions for effective raspberry growing and processing on Lithuanian farms using content and descriptive methods.

### Background of the Literature

Existing studies indicate that practitioners of sustainable agriculture must seek to integrate three main objectives into their work: a healthy environment, economic profitability, and social and economic equity. Every person involved in the food system—growers, food processors, distributors, retailers, consumers, and waste managers—can play a role in ensuring a sustainable agricultural system [9,10,11,12].

The link between healthy people, healthy societies, and a healthy planet puts sustainable food systems at the heart of the European Green Deal, the EU’s sustainable and inclusive growth strategy. It is designed to boost the economy, improve people’s health and quality of life, and care for nature. The European agriculture and food system, supported by the Common Agricultural Policy, is already a global standard in terms of the safety and security of supply, nutrition, and quality. Now, it must also become the global standard for sustainability. Sustainable development is considered a key issue faced in the 21st century [13]. Some studies have pointed out that putting our food systems on a sustainable path also brings new opportunities for operators in the food value chain. New technologies and scientific discoveries, combined with increasing public awareness and demand for sustainable food, will benefit all stakeholders [14,15,16,17,18].

**Figure 1:**
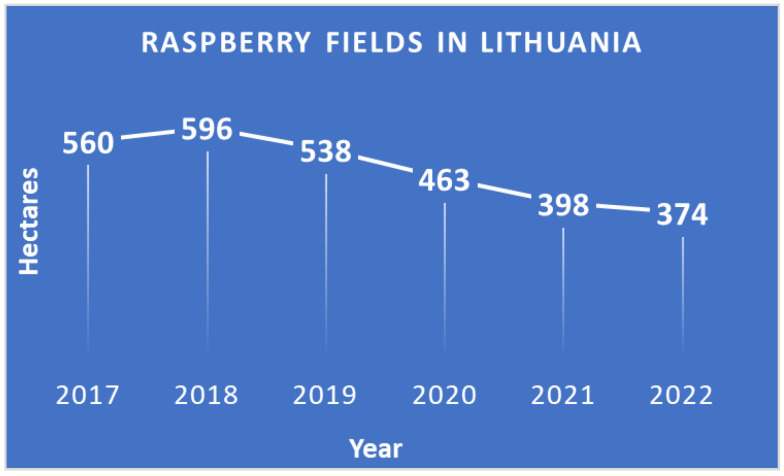
Agricultural land areas declared for raspberry cultivation in Lithuania. Source: State Enterprise Agricultural Data Center.

However, Serbia is struggling with climate problems [19] and Poland is struggling with problems related to the availability of labor during harvesting and low prices of raspberries [20]. There is too little private sector initiative in the field of sustainability in the Lithuanian raspberry market, and insufficient application of sustainable activity solutions in raspberry cultivation and processing on Lithuanian fruit farms. From 2017 to 2022, the declared area of raspberry cultivation in Lithuania decreased from 559.67 ha to 398.37 ha, which is 49%, while the total area around the world is increasing due to the benefits of the plant (Figure 1). Therefore, it can be concluded that the raspberry cultivation farms are unsustainable. And naturally the question arises as to who influences it and what measures should be implemented.

When measuring sustainability, it is usually indicated that it consists of three aspects: environmental, economic, and social factors. However, there are contradictions between these factors because what is good for the economy and brings profit to the farmer, is sometimes unfavorable and negatively affects the environment or people [21,22]. To meet the ever-increasing interest towards agricultural sustainability, many methodologies and tools have emerged, introducing integrated and holistic assessment approaches.

There is still no consensus on the standardization of agricultural sustainability assessment as part of a unified concept of sustainable development. Newly introduced frameworks propose mostly case-specific tools that focus on resource use and their impact on the sustainability of farming practices. In the reviewed studies, stakeholder participation has proved crucial in the determination of the level of sustainability. The effect of resource use and input management is usually the most examined issue in the reviewed studies.

Researchers contribute to the advancement of raspberry growing techniques and sustainable practices. They conduct studies, experiments, and trials to improve crop productivity, disease resistance, and environmental sustainability. The EU’s field-to-fork sustainability targets for fruit and berries cover key processes such as cultivation and primary, secondary, and tertiary processing This food system involves the safe handling (i.e., distribution/transport/supply, storage, and sale/trade) of primary and secondary food products. It also addresses the management of consumer needs and demands as well as broader issues such as minimizing food waste.

In this context, it is essential to demonstrate the contribution of primary and secondary food processing to the wellbeing of the entire food and beverage industry and our economy and environment in general, as well as its contribution to job growth and the improvement of sustainable food production. Numerous studies have revealed that, for sustainability achievement and balanced development, companies must apply strategies and activities that protect, strengthen, and increase human and natural resources for future generations.

Ludwig-Ohm et al.’s (2023) observations that digitization and automation offer great opportunities for horticulture are particularly important. Robotics, innovative sensor-controlled solutions, data management systems, and artificial intelligence can increasingly fulfill complex tasks in the control and management of production systems and help make horticultural production more competitive and sustainable. Thus, digital methods are essential for horticultural production, but their development and use are still in the early stages [23,24].

In a recent study, Liu et al., (2022) pointed out that there is a lack of research on the specific relationship between the digital economy and industrial eco-efficiency, and their study proved that the digital economy has a significantly positive effect on industrial eco-efficiency at the national scale, with diminishing marginal returns [25]. Yang et al., (2022) suggested that managerial relevance is important for decision-makers facing sustainable development challenges [26]. Li et al., (2023) revealed that heterogeneous environmental regulatory instruments (pollution charges and environmental protection subsidies) can jointly improve the green-technology innovation of corporations [27].

Taking into account the importance of digitalization, we have studied and tried to understand what farmers are doing (un)sustainably through our created models. This tool can be used as an example to accelerate management and planning in the raspberry farms. The main goal was to propose sustainable development solutions which can impact the future of the raspberry cultivation and processing industry in Lithuania.

## 2. Research Design

First, we used scientific content analysis to characterize production and identify possible valorization scenarios, sustainability categories, and indicators in growing and processing raspberries. Second, we collected data from raspberry growers and processors about their activities’ sustainability. And lastly, we evaluated the sustainability of raspberry production in Lithuania and presented possible development solutions for improving the sustainability of operations.

In the current article, content and descriptive methods are combined into an original multi-methodology to highlight more than each individual methodology allows. Research results obtained from different methods supplement each other in order to effectively examine sustainability, implementing circular economy principles in raspberry cultivation and processing in Lithuania. In addition, it is important to use a multi-method approach, combining design and research methods with complementary methods in other research fields and disciplines, as this seeks to address complex new research paradigms such as the circular economy, which requires a systemic vision and lifelong thinking. The core of this paper is based on the following research activities: content analysis, data collection, descriptive analysis, and interpretation. The actions performed using the methods are presented in Table 1.

As shown in the table above, the research started with content analysis. A literature review was carried out in March 2023 across the Web of ScienceTM (WOS) and Scopus databases. Conducting a literature review and looking at recent actual documents in this field aims to identify specific research and knowledge gaps, and to discover valuable knowledge and information. In the present paper, the literature survey is completed by investigations into the industrial ground both to obtain new empirical data and to characterize production, identify possible valorization scenarios, sustainability categories, and indicators in growing and processing raspberries, and to test the usability of a proposed method or tool in a raspberry growing and processing environment through real world case studies.

We used content analysis to explore scientific literature and documents and find directions to establish sustainability criteria. This straightforward and very popular method examines the presence, concepts, and subjects in different content formats such as text, image, audio, or video and was chosen to classify important information about the sector into categories and identify key themes and meanings. Using coding text data that was later categorized, we were able to provide valuable insights, making it the perfect mix of qualitative (interview of experts and questionnaire survey assessment of sustainability in farms) analysis.

Secondly, we collected data from raspberry growers and processors. We interviewed nine respondents from the Šiauliai, Klaipėda, Panevėžys, Kaunas, and Alytus districts, and collected farmer-assessed sustainability levels based on their experience and farms’ data through questionnaires. The method of selecting interviewees is probabilistic as the probability of each element of the studied population to be included in the sample is known, and the method of criterion selection is chosen for the formation of the target samples of respondents, which is detailed in Table 2. The selection of respondents was purposeful, and the farms were selected to highlight the meanings discovered. For selected entities, a natural environment is chosen.

The research used a semi-structured personal interview in which the main questions and the entire procedure were planned in advance, but, at the same time, they could be supplemented and simulated during the interview. For research interviews, the questions were structured in such a way that respondents could freely provide formulated answers where neither content nor form is restricted. The survey was conducted in 2023, in the months of April–May, and experts were interviewed directly; two of them were interviewed by phone, and the rest by going to the farm. This method of questioning is superior to others in that the questions that the respondent does not understand can be explained by the researcher.

For qualitative evaluation, a questionnaire was prepared asking farmers to rate the sustainability of raspberry cultivation and processing on a Likert scale. Each item was rated on a five-point Likert scale, which required the participant to choose the indicator’s priority level: 1—very unsustainable, 2—unsustainable, 3—moderate, 4—sustainable, 5—very sustainable. After collecting all the interviewees’ answers, an average was derived, which was used in descriptive analysis and is presented in the results of this study.

The data obtained from the first and second stages were processed using the Microsoft Excel program, divided, and systematized according to three areas of sustainability: economics, environment, and sociology at work. We used the data obtained from the experts for the interpretive explanation, highlighting data by focusing on essentials and grouping individual facts.

Finally, we conducted applied descriptive analysis to examine the occurrences within this particular sector. One of the principal advantages of descriptive analysis lies in the high degree of objectivity and neutrality maintained by the researchers. However, researchers need to exercise extra caution because descriptive analysis reveals various characteristics of the extracted data, and, if these data points deviate from the expected trends, this may lead to significant data distortion. Compared to other quantitative methods, descriptive analysis is considered more comprehensive, providing broader perspectives of an event or phenomenon. It is flexible in its approach, accommodating the use of any number of variables or even just a single variable to conduct the descriptive research. The sort of analysis used in our study is regarded as a superior strategy for acquiring information since it depicts relationships in a natural way while correctly representing the current environment. Because all of the trends are generated through investigating real-life data habits, this analysis is incredibly authentic and human centric. Furthermore, it aids in the identification of factors and the generation of novel hypotheses that may be investigated further through experimental and inferential investigations. The study is appreciated for its low margin of error, as it draws trends directly from the data’s fundamental features [28].

Descriptive analysis: data received from interviewees’ grouping and graphic presentation enabled us to evaluate the sustainability of raspberry cultivation and production development and revealed unsustainable to very sustainable farms aspects. Results from the descriptive analysis allow us to make assumptions and draw conclusions about the sustainability of raspberry growing and processing in Lithuania. Designing and assessing alternative scenarios of raspberry valorization, creating a digital model for management, and improving sustainability can help farmers and processors to identify the feasibility and pathways to move towards a circular economy. However, the assessment of alternative scenarios is challenged by the complex nature of agrifood networks.

## 3. Results and Discussion

### 3.1. Characterization of Production

The aim of the first step was to characterize the production of raspberry plants that have valorization potential and services that can be provided during the cultivation and processing of raspberries that receive additional income, create economic added value, and identify factors influencing the quality of such production, the potential amount, and the factors affecting it.

Raspberries are caneberries from the genus Rubus that have been cultivated as a food and used as a medicine for centuries. Raspberries are categorized as composite fruits and are made up of several ‘drupelets’, each with a single seed. There are both vegetative and fructifying organs in raspberries. Vegetative organs, which are classified as root, stem, and leaf, serve an important function in supporting the life of the individual plant. The fructifying organs, also known as reproductive organs, on the other hand, are critical for the survival and propagation of the species. These include flowers, seeds, and fruits [29]. Harvesting red raspberry leaves for herbal use should be completed before the plant blooms in mid-morning, once the dew has evaporated and while the leaves’ essential oils and flavor are at their peak. Like most herbs, once the plant begins to bloom, the leaves turn bitter.

Because of these properties, farmers cannot collect the leaves during crop time, and they cannot be considered as wasted food. However, after picking the berries, many stems and leaves remain, which can be used as biofuel or raw material for the production of packaging. Failure to use such raw material is considered as a loss of potential production.

Another unique feature is that when growing raspberry seedlings you can not let the berries ripen in order to develop a good root system. Considering these morphological features of the plant, the farmer must decide in advance what will be his main product in the first growing stage:

- berries (3–5 t/ha);
- leaves (data not found);
- seedlings (12,000 pieces/ha);
- stems (data not found).

When growing raspberries, the farmer can provide the following additional services to increase the sustainability of the farm: agrotourism, education, and training.

The plant material is first received from a qualitative and quantitative point of view. Many factors can influence the amount and quality of raspberry raw material grown. The main references to them in scientific publications are as follows: Berries are very fragile and improper handling during or after harvest can make the berries unsuitable for the fresh market. Berries may therefore be better suited for sale in the processed market where value can be added to the product so it can be sold in processed forms. To be sustainable, the farm must produce adequate yields of high-quality raw material for processing high-quality products, be profitable, protect the environment, conserve resources, and be socially responsible in the long term.

Fresh berries suffer from post-harvest losses at the retail level due to their short shelf life, which limits profitability and reduces the sustainability of production by increasing food waste. Accordingly, sustainable production is an important aspect of berry cultivation and farmers must ensure that the quality and nutritional standards are maintained or improved. Smart production systems and advances in agricultural biotechnology are required to meet these challenges. Berries are widely recognized as one of the best food sources, appropriate for eating raw or processed into juice. The remaining material left after extracting the juice is commonly referred to as berry pomace or a press cake, and it contains components such as the skin, stem, and seeds. Berries include a variety of dietary fiber components, including pectin, lignin, cellulose, hemicellulose, and others. Valorization technology is concerned with the long-term transformation of agri-food waste into useful goods. Despite their potential, these wastes are frequently underused, with only limited uses as bio-compost or biofuel [30].

According to the existing research, agri-food wastes can be a valuable source of useful bioactive chemicals. These bioactive chemicals have been scientifically shown to have antioxidant, antiviral, antibacterial, cardioprotective, anti-tumor, and anti-obesity activities [31,32,33]. Notably, significant volumes of waste are produced as a result of the post-processing extraction of pulp needed to make juice, jams, and purees [34]. Bio actives found in the food processing industry waste include dietary fibers, pigments, vital minerals, fatty acids, antioxidant polyphenolic chemicals, and others. The extraction of these value-added chemicals from trash involves the adoption of environmentally friendly and long-term methods.

A person who wants to grow raspberries in Lithuania must register their individual activities according to the economic activity classification A 01.25, and if they want to process them they must register their activities separately according to the product received or the services provided. It is necessary to note that for each product or service providing a higher added value (e.g., education), the farmer must separately register the economic activity, have certificates from the relevant institutions that they can carry out this activity, and relevant qualifications. In Lithuania, most of the farmers grow one-year raspberry shoots, from August to mid-October, referred to as “autumn raspberries”. The plants are grown unsupported, often directly in the ground. The most popular “autumn” cultivars grown in Lithuania are ‘Polka’ and ‘Polana’, which can be conveniently machine-harvested and used in the processing industry mainly for frozen fruit production. Currently, 374 ha of cultivated raspberries are declared in Lithuania.

Within the food processing sector, substantial parts of the raw materials that enter the factory are ultimately traded as by-products. Directly utilizing these streams for food would require alternative (and generally technically more complex) processing than the chains’ primary product. Hence, a large part of these side streams is only poorly valorized for animal feed, technical applications, and fertilizer production (through composting). Higher value applications, however, can increase the total value generation of the food processing chain.

Growing raspberries for processing entails many of the same efforts required for selling fresh fruit wholesale, such as contacting buyers, filling orders, and delivering. Growers who process the products themselves must follow appropriate state and federal sanitation, processing, and labeling regulations. This may include undergoing regular inspections, purchasing stainless steel equipment, and using water treatments. Contact your state or province department of agriculture and/or health department for details.

Value-added products, such as jams and jellies, may generate a higher profit margin than fresh fruit, but the inputs are much greater. Labor costs will be higher, and appropriate cooking tools will be needed, as well as a steady supply of ingredients, jars, labels, shipping boxes, and so forth. Nevertheless, properly processed products provide the advantage of having year-round, quality, locally produced, specialty foods to sell directly to consumers or through wholesalers. Specialty foods are well suited for distribution in tourist destinations such as wine trails, popular parks, and regional fairs.

Bio-based materials can also open new opportunities in product development by providing novel product characteristics and by using biomass for new purposes. Raspberry production from cultivation to zero-waste processing is described in Table 3 below.

Next, we analyzed the factors affecting the quality of fresh raspberries, which are the main raw material, and all other derived, high-value-added products related to raspberries. After analyzing the scientific literature, we found that the quality of obtained fresh raspberries is more influenced by:

1. Cultivation principles.

1.1. Raspberries can be grown in open ground or under cover or in greenhouses. Growing in greenhouses creates optimal conditions for all pests and diseases, which forces farmers to use significantly more pesticides. As a result, significantly lower quality fruits with poorer sensory, chemical, and bioactive substances are obtained.

1.2. Raspberries can be grown intensively or certified (National, local certificated, ecological, biodynamic ‘Demeter international’, other). A higher quality of raw material is always obtained when the production is certified because independent control is ensured, less pesticide is used, biodiversity is ensured, etc.

2. Plant variety.

The quantity and quality of the desired production depends on the variety, chemical composition, biologically active elements, and antioxidant activity [35].

3. Climatic conditions and soil.

The properties of fresh raspberries in particular depend on the climatic zone and the weather conditions of that time. For example, during the rainy season, the sweetness of raspberries will drop significantly. The latter indicator is measured with a refractometer. It will only be about 9 Brix. Therefore, the manufacturer needs to add significantly more sugar to the product at the end so that the usual taste characteristics of the products do not change for the consumer [36,37,38].

Measuring sustainable development in terms of its results is nearly identical to measuring it in terms of the strategies employed to attain those objectives. Assessing sustainability in terms of inclusive well-being goals is essentially to the same as measuring it in terms of inclusive wealth, which serves as the productive basis that allows individuals to achieve those goals. However, both theoretical understanding and real-world experiences show that, when measuring long-term sustainable development, it is generally easier to gauge the stocks of resources that influence it (the means) rather than the flows of goods and services consumed, which constitute its ultimate end, i.e., inclusive well-being.

To sum up the first research stage, it can be concluded that the characteristics of the products made from a raspberry plant have valorization potential; services can be provided during the cultivation and processing of raspberries that create additional income, create economic added value, and identify factors influencing the quality of such production, the potential amount, and the factors affecting it. These results present sustainability indicators for improving the qualitative and quantitative indicators of higher-value raspberry production.

### 3.2. Economic Sustainability

Indicators represent quantitative tools that synthesize and simplify the data which are crucial for the assessment of certain phenomena. They are used in communication, evaluation, and decision-making [39,40]. The presented analysis of sustainable development concept implementation is a general assessment focused on selected aspects, meeting which affects the overall implementation of the sustainable development concept. The selection of variables for the study referred to each of the spheres responsible for sustainable development, i.e., the social, economic, and environmental spheres.

This study was conducted to discuss the perception of raspberry growers and processors, as it is perceived by small farmers in Lithuania. The study identified some criteria and sub-criteria related to the sustainability of raspberry cultivation and processing.

In order to find out the economic sustainability of raspberry cultivation and processing, farmers were primarily asked how much and what quality of produce they produce, what their income is, what it depends on, what their capital is, how and how much the produced produce is sold, and what activities the farmers undertake to develop new products, processes, or services, or improve those that already exist. Economical sustainability categories with explanations and interviewees’ answers are given in Table 4 below.

From this digital model created to measure economical sustainability in raspberry farms, it is clear that the economic dimension includes five main categories and twenty-one subcategories (Table 4) which measure the economic sustainability of raspberry growers and processors, considering both agricultural productivity, profit, capital, realization, and R&D. To measure economic sustainability productivity categories, interviewees highlighted aspects of product quality and generated additional incomes:

<<…*an important measure of productivity and can vary depending on factors such as cultivar, management practices, and environmental conditions*…>>;

(E001, E003)

<<…*monitoring primary production indicators, such as acreage, yield per hectare, and input costs, helps assess the overall performance of raspberry farming operations. Secondary production in the context of raspberries refers to value-added products derived from fresh raspberries, such as processed goods like jams, jellies, juices, or frozen raspberries*…>>.

(Informants E006, E007 and E008)

Another interviewee noticed that:

<<…*recycling raspberry waste, such as stems and other by-products, for biofuel production contributes to sustainability and waste reduction efforts. We obtain about 8 m^3^/ha of stems for biofuel*…>>.

(E005)

<<…*during fresh berry season, about 20 people visit our farm and pick the berries, spend time in nature with their family*…>>.

(E001, E003, E008, E009)

All interviewees underline the importance of crop losses as leaves:

<<*We do not pick and sell the leaves at all due to the high costs, the need for a workforce, and the lack of realization*>>.

(E009)

<<*Raspberry leaves are also of value in various industries, such as herbal teas and natural health products*>>.

(E007, E008)

When measuring the profit category, the interviewees were pessimistic, and everybody agreed that farms are not competitive internationally due to the percentage tax paid compared to other countries that do not apply or apply very low taxes in order to encourage the consumer to buy as much of the produce as is good for his health. Raspberry growers and processors in Lithuania may be subject to other taxes, including corporate income tax, personal income tax, social security contributions, and local taxes. Specific financial figures, and percentages related to profit, taxes, and charity contributions, can vary based on individual business operations, market dynamics, production scale, and other factors. Interviewees justified their argument as follows:

<<*Profit in raspberry growing and processing in Lithuania is influenced by various factors such as production costs, sales revenue, taxes, and charitable contributions. Standard Production Profit is calculated by subtracting total production costs (including labor, inputs, overheads, etc.) from the sales revenue generated from raspberries and related products*>>

(E002, E008)

Some participants referred to:

<<*The specific VAT rates and regulations may vary based on the nature of the products and current tax laws. Gross Profit Margin is a financial metric that indicates the profitability of a business*>>.

(E005, E006)

<<*The price in Lithuania is influenced by the price of imported production from Poland, Ukraine and Serbia with much smaller taxes*>>.

(informants E005, E007, E008)

According to the interviewees, the location of the land for raspberry cultivation and processing and adequate infrastructure, procuring machinery and equipment, are crucial factors. Interviewees present this argumentation as follows:

*<<Ideally, the land should be situated within a reasonable distance (minimum 5 km) from towns or markets to facilitate transportation and access to labor and resources*… (E001–E009); …*adequate infrastructure is essential for raspberry growing and processing operations: access to roads, water sources, electricity, and other necessary utilities* (E003, E004, E007)>>.

Interviewees E002 and E007 argued that:

<<*For efficient raspberry cultivation and processing we need more: tractors, implements (such as plows, harvesters, and sprayers), refrigeration chambers, sorting and packaging equipment, and other tools necessary for field operations and processing activities*>>.

Interviewees E004, E007 and E008 noted: *<< Raspberry growers and processors to assess their specific capital needs based on their business plan, scale of operation, and growth projections>>.*

Realization category refers to the process of selling and distributing the raspberries and raspberry products that have been grown or produced. Food supply chains in raspberry growing and processing can involve both long and short chains. The supply chain includes activities such as packaging, quality control, marketing, and sales to reach the end consumers or buyers. Long supply chains typically include multiple intermediaries, such as wholesalers, distributors, and retailers, before reaching the end consumer.

Interviewees recognized that they realize 100 proc of production through short chains which is very sustainable for the economy. At the same time, the farmers emphasized that they throw away up to 45% of the production because they cannot sell it. They presented some disenchanted views on this issue:

<<*We are forced to throw away about 50% of the production because we have nowhere to sell it*… (E001, 003–007); …*the local market is small. Our product is classified as a luxury product and not everyone can afford to buy it*… (E002, E009); …*local manufacturers do not buy our products because they import cheap products from neighboring countries. We cannot compete with these countries because our products are taxed much higher*… (E008, E009); …*we can’t sell production wholesale because we don’t have the necessary quantities or can’t ensure the realization for several years to come for suppliers*… (E005, E007, E008)>>.

Some interviewees justified their arguments as follows:

<<…*exporting raspberries requires compliance with international quality standards, proper packaging, and adherence to import regulations of target countries*… (E001, E003–E005); …*marketing includes creating a strong brand presence, advertising campaigns, participation in trade shows or exhibitions, online marketing, and social media engagement to create awareness and demand for the raspberries and raspberry products*… (E008)>>.

This shows that building relationships with buyers, understanding consumer preferences, and staying informed about market trends can contribute to successful realization strategies in the raspberry industry in Lithuania.

Another research and development (R&D) category involves the exploration and implementation of various techniques, technologies, and investments in human resources. The interviewees placed emphasis on the advancements in irrigation systems, precision farming methods, disease and pest management techniques, soil fertility management, and post-harvest handling technologies:

<<…*R&D activities emphasize the adaptation of techniques and technologies to the specific climatic, soil, and environmental conditions in Lithuania* (E004); …*R&D initiatives often involve collaboration between research institutions, universities, agricultural extension services, and industry stakeholders. Best practices, and joint research projects aimed at addressing specific challenges in raspberry growing and processing. These investments enhance the expertise and skills necessary to conduct R&D and drive innovation in raspberry cultivation and processing*… (E001, E004); …*government grants, research programs, and agricultural development initiatives provide financial support for R&D projects* E002, E005, E008>>.

According to interviewees E007 and E009, industry collaborations, partnerships, and cooperative research efforts contribute to the availability of resources for R&D activities. Interviewee E004 noted that:

<<*These efforts contribute to the development of sustainable practices, the adoption of advanced technologies, and the continuous improvement of raspberry cultivation and processing methods*>>.

Evidence from the interviews suggests that product quality, income from additional activities, and realization through short chains are the sustainable sides of the farms. According to the interviewees, farmers do not use the full potential of the grown production, e.g., they do not collect leaves, they pay high taxes compared to neighboring countries, and they do not realize a lot of production. These areas are inefficient and unsustainable on farms. From all the economic subcategories, assuming that each of them is rated with the highest rating of five points, they could score a maximum of 100 points. Lithuanian raspberry growers rated their activity 53.11 points out of 100 possible which is 53.11%. This shows that there are issues to be addressed in the sector.

In recent years, other scientists have increased their emphasis on the relationship between the economy and sustainable development. Milic et al. (2013) pointed out that the increased economic efficiency of raspberries can be achieved from its primary production, as well as its processing and improved product quality. The economic importance of raspberry consists of a relatively large amount of profit per unit of invested capital and labor [49]. Pantic et al. (2017) proposed that the growth, profitability, and competitiveness of the sector must be improved through investments in all phases (production, processing, and distribution) and changes in the export structure [50]. Qattan et al. (2021) analyzed economic factors influencing the supply and demand of raspberries. They underlined as a general conclusion that macroeconomic indicators are very important factors in the success of production, but also in the demand for raspberries. In addition, they noticed that exporting unprocessed raspberries is lucrative, but it is even more profitable to export raspberry-based products [51]. Our research reveals that manufacturers in Lithuania pay very little attention to wholesale trade and export. However, it is a positive thing that investments are made in the processing of high-value products.

### 3.3. Environmental Sustainability

To find out the environmental sustainability of raspberry cultivation and processing, farmers were asked what the fertility of their land is, whether they do land surveys, how often, whether they know the damage caused by soil, wind, and water erosion to their farm, how much and what quantities of fertilizers and pesticides they use, how they determine what quantity to use, what quality and varieties their seedlings are, whether they use bio-increase measures, how much and what kind of water and energy they use for the farm, how accounting is kept, what cultivation and processing principles are applied on the farm, who controls the quality, and what the emissions from growing and processing their raspberries are. Environmental sustainability categories with explanations and interviewee answers are given in Table 5.

Another important environmental sustainability measurement dimension was divided into four categories: soil, plant, other resources, and emissions. The majority of interviewees recognized that soil plays a crucial role in determining the success and sustainability of the crops. Land performance score is a measure of soil quality and fertility, taking into account various factors such as nutrient content, organic matter levels, pH, drainage, and soil structure. Maintaining soil fertility is essential for optimal raspberry growth. This involves balancing nutrient levels through practices like soil testing, organic matter additions (such as compost or manure), and targeted application of fertilizers based on crop requirements. Encouraging beneficial soil organisms such as earthworms, bacteria, fungi, and other microorganisms helps enhance soil structure, nutrient cycling, and overall soil health. Measuring the environmental sustainability soil category, interviewees presented some disenchanted views on this issue:

<<…*an important measure of productivity and can vary on many factors*… (E001); …*soil is an ever-changing system*… (E002); …*in our area, erosion and parent material, soils vary very much*…(E006); …*it is important to minimize compaction by avoiding excessive machinery traffic on wet soils, utilizing appropriate tire inflation pressures, and implementing controlled traffic farming techniques*… (E009); …*raspberry fertilizing needs are very basic and not hard to keep up with*… (E008)>>.

Interviewees underlined some traits of erosion:

<<…*in raspberry growing and processing in Lithuania, cover crops help prevent erosion, improve soil structure, suppress weeds, and increase organic matter content*…>>;

(E002)

<<…*implementing field edges, hedgerows, or buffer zones with diverse plant species can provide a habitat for beneficial insects, pollinators, and wildlife*…>>;

(E005)

Another interviewee added:

<<…*implementing erosion control measures such as contour plowing, terracing, windbreaks, and vegetative cover helps minimize soil erosion caused by water and wind, preserving soil fertility and reducing sediment runoff into water bodies*…>>

(E008)

All participants recognized that:

<<…*farmers don’t know how detected erosion damage and how this could be done*…>>.

(E001–E009)

Interviewees focused more of their attention on fertilizers and chemical management:

<<…*assessing crop nutrient requirements through soil testing and adopting precision fertilization techniques can optimize fertilizer use and minimize environmental impacts. Specific data on the amount of fertilizer used, pesticides, and chemicals applied in raspberry growing and processing in Lithuania may vary depending on individual farm practices, crop conditions, and adherence to sustainable agricultural standards and regulations*… (E006); …*fertilizers are an essential tool to help us to grow a good crop and to be competitive*… (E004, E007); …*the intensity of use of chemical fertilizers and pesticides is several times higher than ten years ago. However, the efficiency of agrochemical use is low*… (E003, E009)>>

Everybody agreed that:

<<…*the accounting of fertilizers and pesticides is very strictly controlled by state institutions, and we waste a lot of time in inspections*… (E001–E009); …*chemical pesticides and fertilizers are important for sustaining and boosting our production*… (E001–E009)>>.

Interviewees justified their arguments as follow:

<<…*the prices of fertilizers and pesticides are very high, so farmers have to calculate the required amount very carefully*… (E002, E005, E008); …*chemical fertilizers allow farmers to increase their yields, but using only chemical fertilizers without organic or biological fertilizers makes the soil unproductive and less profitable. To solve this problem, the state promotes and supports production that uses less fertilizer and is ecological. Using support, we grow better quality berries*… (E003, E007–E009)>>.

Another important element of environmental sustainability is the plant itself: its variety and quality. Interviewees illustrated such points of view about this element:

<<…*in Lithuania, several raspberry varieties are cultivated, taking into consideration factors such as high-quality planting material, resistance to pests and environmental conditions, and adaptation to the region. It’s important for farmers to select raspberry varieties that are well-suited to local conditions, resistant to prevalent pests and diseases, and capable of thriving in the Lithuanian climate*…>>

(E002, E007)

<<…*consulting with local agricultural extension services, nurseries, or raspberry experts can provide valuable guidance on the most suitable varieties for specific regions in Lithuania. Promoting biodiversity in raspberry cultivation is essential for maintaining ecosystem resilience and sustainability. By incorporating a diverse range of plant species, farmers can provide habitats for beneficial insects, pollinators, and other wildlife*…>>.

(E004)

Some interviewees highlighted other such aspects:

<<…*water use and energy use are important resources to consider in raspberry growing and processing in Lithuania. Techniques such as drip irrigation or micro-sprinklers can deliver water directly to the root zone, minimizing water loss through evaporation and improving water efficiency*… (E002); …*utilizing soil moisture sensors or weather-based irrigation systems can aid in efficient water management*… (E003); …*installing rainwater harvesting systems can help reduce reliance on groundwater or municipal water sources, especially during periods of low rainfall*… (E007); …*treating and reusing process water for non-potable purposes such as irrigation or cleaning can contribute to water conservation efforts. Generating on-site renewable energy can offset energy consumption and contribute to environmental sustainability*… (E008)>>.

Regarding the emissions in raspberry cultivation, the farmers had no knowledge, and this is reflected in their answers:

<<…*implementing sustainable farming practices can help reduce emissions in raspberry cultivation. This includes minimizing the use of synthetic fertilizers and pesticides, adopting organic farming methods, practicing crop rotation, and optimizing irrigation techniques to minimize water usage*…>>;

(E005)

<<…*implementing recycling and composting programs, minimizing food waste, and using by-products for animal feed or other value-added products can contribute to waste reduction and lower environmental impact*…>>.

(E008)

Interviewee E009 agreed that:

<<…*efficient transportation and logistics strategies can help reduce emissions associated with the distribution of raspberries*…>>.

From the results of the interviews and explanations mentioned above, it can be concluded that using energy-efficient machinery and equipment, optimizing lighting systems, and implementing insulation measures make it possible to conserve energy. By focusing on water conservation and efficient energy use, raspberry growers and processors in Lithuania can reduce their environmental impact, lower their operational costs, and contribute to sustainable agricultural practices. Farmers adhering to recognized sustainability certifications and standards, such as organic certifications, can ensure that raspberry production meets specific environmental criteria. Investing in research and innovation can lead to the development of new technologies and practices that further reduce emissions in raspberry growing and processing.

From all the environmental sub-categories, assuming that each of them is rated with the highest rating of five points, the farmers could score a maximum of 55 points. Lithuanian raspberry growers rated their activity as 25.78 points out of a possible 55, which represents 51.56%. This shows that there are issues to be addressed in the sector. Interviewees argued that soil erosion, undetermined soil properties, and biodiversity are the least sustainable areas, but cultivation and processing principles and high land performance score are the most sustainable environmental areas.

Comparing environmental sustainability in raspberry growing and processing with other studies, our results show that cultivating and processing principles are the strongest areas with, farmers adhering to recognized certifications and standards, such as organic certifications which ensure that raspberry production meets specific environmental criteria. Krishkova et al. (2020) [61] pointed out that improving the economic performance parameters of raspberry production can be achieved through the optimization of alternative agricultural management practices. Vasquez-Ibarra et al. (2021) revealed that the variability in the environmental categories can be associated with three main causes: the quantity of agrochemicals used, the type of agrochemicals, and the yield obtained in each orchard [62]. These causes can individually or jointly influence the variability in the environmental impact categories, as is the case of the quantity of agrochemicals applied and yield obtained. Fertilizers make the greatest contribution to the environmental impact categories studied, followed by pesticides, and pruning waste management. Our study revealed that soil fertility and pest and agricultural management are the weakest areas in raspberry growing in Lithuania.

### 3.4. Social Sustainability

In the third part of the interview, on social sustainability, farmers were asked about their how work functions and their responsibilities, and the specifics of well-being, safety, and comfort in the workplace. Social sustainability categories with explanations and interviewees answers are given in Table 6.

The last and third digital model for measuring social sustainability in raspberry farms is divided into five main categories. Interviewees primarily distinguished two completely different categories of workers on the farm. They detail the job functions and responsibilities of farm owners:

<<…*farmers are the primary individuals responsible for owning and managing the raspberry farms. They make decisions related to cultivation practices, and overall farm management. There are responsible for inspecting and ensuring the quality of raspberries at different stages of production*… (E002, E006); …*farmers coordinate shipping logistics, track inventory, and ensure timely delivery. They develop marketing strategies, manage customer relationships, negotiate contracts, and work to expand the market reach and demand for raspberries*… (E001, E004) …*family members in many cases, family members of the farmers actively participate in the raspberry cultivation process. They contribute to tasks like planting, weeding, harvesting, and other farm activities*… (E008); *Family involvement often strengthens the sense of ownership and continuity of the farm operation*… (E005)>>.

Meanwhile, differences in salaried or seasonal workers are highlighted by these interviewees as follows:

<<…*field workers assist farmers during the various stages of raspberry cultivation, including planting, weeding, pest management, and harvesting. Field workers often work on a seasonal or temporary basis, especially during peak farming*… (E002); …*employees working in raspberry processing plants are responsible for transforming harvested raspberries into various products*… (E005, E009); …*seasonable workers may be involved in sorting, washing, packaging, operating machinery, and producing value-added products such as jams, juices, or frozen raspberries*… (E003, E006, E008).>>

It is important to note that the size and scale of raspberry farms and processing facilities can vary, which influences the number and composition of employees. According to one interviewee:

<<…*field workers provide crucial support to farmers during peak seasons, such as during planting and harvesting*… (E007); *workers assist in tasks like transplanting young raspberry plants, weeding, mulching, and maintaining irrigation systems. During harvest time, field workers carefully pick the ripe raspberries, ensuring quality and proper handling*… (E008); *employees involved in logistics and distribution manage the transportation and delivery of raspberries from farms or processing plants to markets or retailers. They ensure proper packaging, storage, and handling of raspberries to maintain product freshness and quality throughout the supply chain*… (E003)>>

Talking about well-being on the raspberry farms, interviewees emphasized the owners and their family members undertaking training and education:

<<…*farmers and workers can acquire knowledge about agricultural practices, plant biology, soil management, pest control, and more*…>>;

(E003, E006)

Another noted that:

<<…*providing proper training, ensuring the use of safety equipment, implementing safe working procedures, and regularly inspecting machinery and equipment are essential to prevent accidents and injuries*…>>.

(E004)

And another interviewee continued that:

<<… *providing ergonomic tools, training on correct posture and lifting techniques, and scheduling breaks can help minimize the risk of injuries and promote the well-being of workers. Raspberry processing often involves the use of machinery, such as sorting, washing, and packaging equipment. Ensuring proper maintenance, operator training, and safety guards on machinery are necessary to prevent accidents and injuries*…>>

(E008)

Others illustrated their point of view on work stress, safety, and physical demand:

<<…*raspberry growing involves physical labor, which can contribute to overall fitness and well-being*… (E004); …*farmers and field workers engage in activities such as planting, pruning, weeding, and harvesting, which provide exercise and promote physical health*… (E001, E007); …*working in raspberry fields allows individuals to connect with nature and experience the benefits of spending time outdoors. Being in natural environments has been shown to improve mental well-being, reduce stress, and increase feelings of calmness and relaxation*… (E002); …*seeing the fruits of their labor grow and contribute to the production of a valuable crop can enhance job satisfaction and overall well-being*… (E008); …*raspberry growing often involves working in teams or within a community of farmers. This fosters social connections, cooperation, and a sense of belonging*… (E009)>>.

Creating a comfortable workplace environment for workers involved in raspberry growing and processing in Lithuania is crucial for their well-being and productivity. Some participants referred to the workplace comfort:

<<*we ensure that workers have access to clean water, restroom facilities, and handwashing stations… (E003); …we implement safety controls such as emergency response protocols, fire safety measures, and proper ventilation systems*… (E005), …*farmers control noise levels by implementing soundproofing measures or providing personal protective equipment (e.g., earplugs)*… (E006); …*we try to optimize lighting conditions, utilizing natural light or providing appropriate artificial lighting to minimize eye strain. Address vibration hazards associated with machinery or equipment to prevent long-term health issues. Provide appropriate personal protective equipment (PPE) and ensure workers are trained on proper handling, storage, and disposal procedures*… (E008); …*farmers implement strict protocols for chemical handling, including labeling, safety data sheets, and regular monitoring and provide support mechanisms, such as employee assistance programs or counseling services, to address physical and mental health needs*… (E009); …*farmers offer free meal breaks or subsidized meal options to support nutrition and well-being*… (E001)>>.

From the results of such an analysis, it can be concluded that it is important to address safety concerns in raspberry growing and processing in Lithuania to ensure the well-being of workers and consumers. Raspberry farming involves physical labor and operating machinery, which can pose risks to workers’ health and safety. Collaborative work environments can contribute to positive social interactions, support networks, and a sense of community well-being.

To address these safety concerns, it is essential to have regulatory frameworks in place, enforce compliance with safety standards, provide training and education to workers, and conduct regular inspections and audits to ensure adherence to safety protocols. Collaboration between government agencies, industry stakeholders, and workers’ organizations can play a significant role in promoting safety in raspberry growing and processing in Lithuania. It is important to align these workplace comfort initiatives with applicable labor laws and regulations in Lithuania to ensure compliance and the well-being of workers. Regular assessments, feedback mechanisms, and continuous improvement efforts can help monitor and enhance the comfort and safety of the work environment.

The third social sustainability measurement dimension was divided into five categories: worker categories, working conditions, well-being, safety, and workplace comfort. From all the environmental sub-categories, assuming that each of them is rated with the highest rating of five points, they could score a maximum of 75 points. Lithuanian raspberry growers rated their activity 54.11 points out of 55 possible, which comes to 72.15%. This social dimension was rated by the highest percentage of farmers as the most sustainable. Finally, it is worth mentioning that there are no previous studies that analyze the social sustainability of raspberry growing and processing.

All the participants agreed that permanent jobs for farmers and their family members, training, and education for them according to the need, and working hours for seasonable employees are the most sustainable socio-areas. However, working hours for owners/farmers last too long and they often go without remuneration. Their profit depends on lots of factors (e.g., weather, prices) and workplace comfort depends on severe weather conditions. The sustainability of raspberry cultivation and processing in Lithuania, rated on a Likert scale from very unsustainable to very sustainable, is presented in Table 7.

The research data revealed how sustainability is evaluated by nine farmers according to the established categories. Disclosed unsustainable categories such as production loss in the growing stage, high taxes, bad product realization, undetermined soil properties and erosion, lack of biodiversity, unlimited working hours with profit depending on severe weather conditions or political decisions and workplace comfort are directions for sustainable activity solutions in raspberry growing and processing on Lithuanian farms. Sustainable intensification targets go beyond production, environmental, economic, or social performance.

Raspberry growers should improve the collection and sale of produced raw materials (e.g., leaves), thus contributing to zero-waste technologies and reducing food waste. In addition, the lack of realization also affects competitiveness on an international scale; therefore, scientific research and analysis of laws and documents are necessary, which would reveal what measures at the state level would improve the situation in this sector. In the field of environmental protection, the systematicity of conducting soil tests should be improved. Training on how to calculate the amount of fertilizer based on the results of the soil tests would help farmers save money, and the structure of the soil would improve. In the area of social sustainability, risk management factors such as business insurance should be improved in order for farmers to experience less stress due to the quantity or quality of the produce they grow.

The strengths of sustainability show that raspberry growers and processors have a good potential for the development and realization of high-quality products and they meet the requirements of today’s consumers (naturalness, ecology, etc.). The principles of cultivation and processing are of high standards and are friendly to the environment. Good working conditions for workers show that farmers are trying to attract labor, but do not use all available means for continuity of activity in this area, such as support for communities, involvement in local traditions, and events supporting them.

The results of the qualitative study show that product quality, income from additional activities, product realization through short food chains, cultivation and processing principles, permanent jobs for farmers and their family members, training and education for them according to the needs, and working hours for seasonable employees are the most sustainable directions in raspberry growing and processing for effective transformation. Disclosed unsustainable categories such as production losses in the growing stage, high taxes, bad product realization, undetermined soil properties and erosion, lack of biodiversity, unlimited working hours with profit depending on severe weather conditions or political decisions, and workplace comfort are directions for sustainable activity solutions for raspberry growing and processing on Lithuanian farms. Although the results of the study do not show the situation of the entire population in the sector, they can be used for further research, and raspberry growers can use it as a digital model for the sustainability, efficiency, and developmental directions of their farm. Additional policy efforts are needed to manage sustainability in the berry sector.

## 4. Conclusions

The results of a comparative analysis of the scientific literature determined the sustainability of raspberry production, including potential quantities from cultivation to zero-waste processing, and described the factors influencing the quality of primary raspberry cultivation. This aspect is crucial for valorization and the creation of high-quality products. We identified and selected sustainability indicators for improving the qualitative and quantitative raspberry production and revealed sustainable development solutions in three dimensions: economic, environmental, and social, which can improve raspberry growing and processing in Lithuania.

The results from our qualitative study revealed raspberry cultivation and processing sustainability factors in Lithuania on a Likert scale from very unsustainable to very sustainable in the economic, environmental, and social dimensions. Therefore, the criteria presented in Table 4, Table 5 and Table 6, as well as the created model, could be a great start for business self-assessment, and setting future goals for sustainable business.

By integrating the above-mentioned sustainable development solutions in three dimensions, raspberry growing and processing in Lithuania can contribute to environmental conservation, economic growth, and social well-being, fostering a more sustainable and resilient agricultural sector. By integrating cultivation and processing principles, raspberry growers and processors in Lithuania can work towards reducing emissions, mitigating climate impact, and contributing to a more sustainable and environmentally friendly raspberry industry. By investing in R&D, Lithuania can enhance its raspberry industry’s competitiveness, improve productivity, and address emerging challenges.

## Figures and Tables

**Table 1:** Research methods and processes applied in the present paper.

Methods	Actions	Data and Information Used	Date
STAGE 1 Content analysis	4.1. Performing exhaustive review, analysis, synthesis of the literature and documents, grouping, and comparison 4.2. Characterizing production and identifying possible valorization scenarios 4.1. Identifying sustainability categories and indicators in growing and processing raspberries	Web of ScienceTM (WOS) and Scopus databases, media, project results, laws, EU and Lithuanian strategic documents	March 2023
STAGE 2 Data collection	Collecting data from raspberry growers and processors: 4.1. Interview based on a semi-structured questionnaire. 4.2. Farmers evaluation of farm’s sustainability	Interviewees’ answers	April–May 2023
STAGE 3 Descriptive analysis	4.1. Evaluating the sustainability of raspberry cultivation and production development for digital model 4.2. Presenting raspberry growing and processing sustainability factors in five levels from low to high	Farmers answers from questionnaires	June–July 2023

**Table 2:** Interviewers’ data.

Criteria for the Selection of Respondents		Interviewers								
		E001	E002	E003	E004	E005	E006	E007	E008	E009
**Area of cultivated** **raspberries**	1–2 ha			+	+			+		+
	2–3 ha	+	+				+			
	3–20 ha					+			+	
**Raspberry processing**	Primary processing	+	+	+	+	+	+	+	+	+
	Secondary processing					+			+	
**Experience in cultivation and** **processing**	From 3 to 5 years			+	+		+	+		
	From 5 to 10 years									+
	10 years and more	+	+			+			+	
**Number of permanent employees**	Up to 2			+	+		+	+		+
	From 2–5	+	+			+			+	
	More than 5									
**Number of seasonable workers**	Up to 2									
	From 2–5						+			
	More than 5	+	+	+	+	+		+	+	+

**Table 3:** Raspberry production from cultivation to zero-waste processing.

**0 WASTE ← CULTIVATION**	**Activity Sector**	**Main Production**	**Possible Quantity of Production from 1 ha**	**Additional Products or Services Available**
	1. Raspberry cultivation	Fresh raspberries (raw material for processing)	Raspberries—2, 6 t	Agrotourism and educational services. Leaves, stems
	2. Food and drink	Frozen raspberries, juices, jams, wine, dry products, etc.	Juice—87 proc. Seeds—12–13 proc.	Seeds
	3. Cosmetics	Oils, seeds after extractions, extracts	Aprox. 14 proc of oil from dry seeds (8–14% humidity)	Seeds after extractions for biofuel, animal feed
	4. Pharmacy	Food supplements, vitamins (ex. E), omega acids, dietary fibers, etc.	Vitamin E 0.87 mg/100 g fresh berries [xx]; α-Linolenic (ω − 3) 37.7 g/100 g	Seeds after extractions for biofuel, animal feed
	5. Other industries	In perspective—packaging, micronutrient fertilization	No scientific or practical data found *	Biofuel

* Additional research is needed.

**Table 4:** Digital model for measuring economical sustainability in raspberry farms.

Sustainability Measurement Category	Subcategory with Explanation	Results from Informants’ Open Questions	Intervieweers Data	Score Average
**1. Productivity**	1.1. Fresh raspberry yield can be from 1.5 to 6.15 t/ha	1.4–2.3 t./ha.	E001, E003 E005, E008	2.78
	1.2. Leaves yield. No data found	0	E001–E009	1.00 **
	1.3. Production quality can be from category III to I (best)	1 category	E001–E009	4.89 *
	1.4. Valeur/price is 1.2–16.50 USD/kg, average—6 USD/kg	3.00 eur/kg	E001–E009	2.56
	1.5. Secondary production output subcategory is not set.	About 500–750 kg puree	E005, E008	3.44
	1.6. Third production output subcategory is not set.	Oil 3 L	E008	1.67
	1.7. Recycled waste on the farm	9 m^3^ of stems for biofuel	E005	1.56
	1.8. Other services (education, training, agritourism). The subcategory is not set	2 events	E002, E006	3.33
		Agritourism for average 20 people	E001, E003, E008, E009	4.33 *
**2. Profit**	2.1. Standard production profit EUR per 1 ha—1028	Profit after taxes 700–1000	E002, E005, E006, E008	1.22 **
	2.2. VAT taxes—lowest—0% (Poland, Ireland, Malta), 5%—Latvia. The highest—27% (Hungary)	High VAT taxes—21 proc.	E001–E009	1.00 **
	2.3. GPM, other taxes	GPM—15 proc.	E001–E009	1.22
**3. Capital**	3.1. Location of a land (close to town min 5 km)	Location is not good	E004, E007	2.44
	3.2. Infrastructure	Bad infrastructure	E003, E004	2.00
	3.3. Berries are grown on own land/lease from private individuals/lease from the state.	Own land	E001–E009	2.44
	3.4. Procurement of machinery and equipment (tractor, implements, refrigeration chambers, etc.)	Average. Missing digitized technologies	E002; E004, E005, E008	3.33
**4. Realization**	4.1. Realization in proc. from the received production (grown or produced).	From about 50 to 80 proc.	E001–E009	1.44
	4.2. Food supply chains (long and short)	Realisation through short supply chains 100%	E001–E007	4.22 *
	4.3. Wholesale and export	20% from all production	E008, E009	1.56
**5. R&D**	5.1. Techniques and technologies used	Minimum or not at all	E001–E009	2.44
	5.2. Investments in human resources	Minimum or not at all	E001–E009	3.33
	**TOTAL ECONOMICAL SUSTAINABILITY SCORE: 53.11/100**			

* Most sustainable, ** Least sustainable. The economical sustainability categories in the table are prepared based on the following literature sources [34,41,42,43,44,45,46,47,48].

**Table 5:** Digital model for measuring environmental sustainability on raspberry farms.

Sustainability Measurement Category	Subcategory with Explanations	Results from Informants Open Questions	Interviewees Data	Score Average
**1. Soil**	1.1. Land performance score	38 to 50 points	E001–E009	4.11 *
	1.2. Soil fertility: amount of organic carbon and humus (organic matter) in the soil; Contaminants, the amount of nutrients and salts; Biodiversity in soil; Covering crops, Soil compaction, acidity; Landscape heterogeneity	Soil properties are not determined	E001–E009	1.33
	1.3. Soil, water, and wind erosion	Not determined	E001–E009	1.00 **
	1.4. Amount of fertilizer used t/ha	Fertilizers are used at low rates without soil testing	E001–E004 E007, E008	3.33
	1.5. Pest and agricultural chemical management. Amount of pesticides and chemicals used l., kg/ha	Farmers use pest and disease prevention and weed control measures. Strict state control	E001–E009	1.44
**2. Plant**	2.1. Variety. High quality planting material, resistant to pests and environmental factors, adapted to the region	Cultivate primocane varieties Polka, Kwanza, Kweli	E001–E009	2.56
	2.2. Biodiversity	No biodiversity measures	E001–E009	1.22 **
**3. Other resources**	3.1. Water use	Rarely use irrigation system. Pay taxes	E001, E002, E005, E008	3.44
	3.2. Energy use	Use their own green energy	E002, E008	1.56
**4. Emissions**	4.1. Cultivation and processing principles	Sustainable cultivation system	E002–E009	**4.78**
	4.2. Carbon foodprint	Does not count	E001–E009	1.00 **
**TOTAL ENVIRONMENTAL SUSTAINABILITY SCORE: 25.78/55**				

* Most sustainable, ** Least sustainable. The environmental sustainability categories in the table are prepared based on the following literature sources [52,53,54,55,56,57,58,59,60].

**Table 6:** Digital model for measuring social sustainability on raspberry farms.

Sustainability Measurement Categories	Subcategories with Explanations	Results from Informants Open Questions	Interviewees Data	Score Average
**1. Worker categories**	1.1. Farmer and his family members	Permanent job, up to two	E001–E009	4.89 *
	1.2. Employee	Seasonable up to five per 1 ha	E001–E009	2.11
**2. Working conditions**	2.1. Remuneration and overtime payment	*Farmer*: Payed from annual profit	E001–E009	1.67
		*Employee*: Minimum		3.44
	2.2. Working hours	*Farmer*: 48 h per week and more	E001–E009	1.33
		*Employee*: 40 h per week or less	E001–E009	5.00 *
**3. Well being**	3.1. Facilities to help during the work, rights, and benefit	Fully equiped	E001, E002, E005, E008	4.22
	3.2. Training and education.	*Farmer*: according to the need	E003, E006	4.78 *
		*Employee*: none	E001–E009	1.11 **
	3.3. Work stress	*Farmer*: high level	E001–E009	1.22 **
		*Employee*: none	E003, E009	4.11
	3.4. Physical demand	The job requires good physical condition	E001–E009	2.44
**4. Safety concerns**	4.1. Personal protecting tools	Fully equipped	E001–E009	3.78
	4.2. Warnings, instructions, and others displayed in the workplace	Everything is clearly indicated	E001–E009	3.44
	4.3. Accident data, expenditure on illness, and accident prevention	Very rare and without serious consequences	E001–E009	3.33
**5. Workplace comfort**	5.1. Facilities to help workers during the work, controls, displays, and proper support	Tools that facilitate work are optimized and used as much as possible	E003, E005, E006, E008, E009	3.44
	5.2. Temperature, noise, lighting, and vibration	Depends on weather conditions in the fields and…	E001–E009	1.11 **
	5.3. Toxic or radioactive chemicals or other hazards	All farmers work with pesticides, herbicides, etc.	E001–E009	1.22 **
	5.4. Social investments and principles (e.g., coffee makers, free meal breaks, sports, and activities, vacations, insurance, etc.)	Organize holidays, make free meal beaks	E002, E005, E007, E008	1.44
**TOTAL SUSTAINABILITY SCORE: 54.11/75**				

* Most sustainable, ** Least sustainable. The social sustainability categories in the table are prepared based on the following literature sources [63,64,65,66,67,68,69,70,71,72].

**Table 7:** Results from digital models for measuring sustainability of the raspberry farms.

	Unsustainable	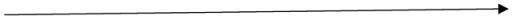			Sustainable
	1	2	3	4	5
**Economy**	Productivity—no leaves yield, low profit after taxes, high taxes, bad realization, a large amount of wasted food, no high tertiary processing.	Productivity—recycled waste, GPM taxes. Capital—location, infrastructure, land ownership. Realization—wholesale and export. R&D—techniques and technologies used	Productivity—fresh berries, primary and secondary production processing, other services. Capital—procurement of machinery and equipment. R&D—investments in human resources	Productivity—agrotourism and other services like education, events. Almost 100 percent of the production is realized through short food chains	Productivity—high production quality, production is certified, farmers participate in food quality schemes, processes are controlled by state institutions.
**Environment**	Soil fertility plays a crucial role in determining the success and sustainability of the crops. Pest and agricultural management. Biodiversity	No green energy use. Generating on-site renewable energy can offset energy consumption and contribute to environmental sustainability	Amount of fertilizer used should be assessing crop nutrient requirements through soil testing and adopting precision fertilization techniques. High plant quality and good variety. Accounting and control of water consumption.	Soil—land performance score. A measure of soil quality and fertility, taking into account various factors such as nutrient content, organic matter levels, pH, drainage, and soil structure.	Cultivation and processing principles. Farmers adhering recognized certifications and standards, such as organic certifications which ensure that raspberry production meets specific environmental criteria
**Social**	Well-being—no training and education for seasonable employees. High stress level for farmers. No social investments and principles. It could secure the workforce in the future.	Working conditions—remuneration and working hours for farmers. Well-being—job requires good physical condition. Workplace comfort—depends on weather conditions, farmers work with pesticides, chemicals, etc.	Working conditions—remuneration for seasonable workers. Workplace comfort—facilities to help during the work. Collaborative work environments can contribute to positive social interactions, sup-port networks, and a sense of community well-being.	Well-being—facilities. Safety—fully equipped personal protecting tools, warnings, instructions, and others clearly indicated, accidents are very rare and without serious consequences.	Worker categories—farmer. Working conditions—working hours for seasonable employees. Well-being—training and educations for farmers.
